# Retracted: Antitumor Molecular Mechanism of Chlorogenic Acid on Inducting Genes GSK-3*β* and APC and Inhibiting Gene *β*-Catenin

**DOI:** 10.1155/2021/5414158

**Published:** 2021-01-25

**Authors:** Journal of Analytical Methods in Chemistry

*Journal of Analytical Methods in Chemistry* has retracted the article titled “Antitumor Molecular Mechanism of Chlorogenic Acid on Inducting Genes GSK-3*β* and APC and Inhibiting Gene *β*-Catenin” [1]. Confidential information from Sichuan Jiuzhang Biological Science and Technology Co., Ltd. (previously known as Sichuan Jiuzhang Biochemical Engineering Science and Technology Development Co., Ltd.) was published without permission, as confirmed by a court judgment. A previous corrigendum that acknowledged the involvement of the company was not sufficient [2].
